# Diagnostic Accuracy of a Mobile AI-Based Symptom Checker and a Web-Based Self-Referral Tool in Rheumatology: Multicenter Randomized Controlled Trial

**DOI:** 10.2196/55542

**Published:** 2024-07-23

**Authors:** Johannes Knitza, Koray Tascilar, Franziska Fuchs, Jacob Mohn, Sebastian Kuhn, Daniela Bohr, Felix Muehlensiepen, Christina Bergmann, Hannah Labinsky, Harriet Morf, Elizabeth Araujo, Matthias Englbrecht, Wolfgang Vorbrüggen, Cay-Benedict von der Decken, Stefan Kleinert, Andreas Ramming, Jörg H W Distler, Peter Bartz-Bazzanella, Nicolas Vuillerme, Georg Schett, Martin Welcker, Axel Hueber

**Affiliations:** 1 Institute for Digital Medicine University Hospital Giessen-Marburg Philipps University Marburg Marburg Germany; 2 AGEIS Université Grenoble Alpes Grenoble France; 3 Department of Internal Medicine 3 Friedrich-Alexander-University Erlangen-Nürnberg and Universitätsklinikum Erlangen Erlangen Germany; 4 Deutsches Zentrum für Immuntherapie (DZI) Friedrich-Alexander-University Erlangen-Nürnberg and Universitätsklinikum Erlangen Erlangen Germany; 5 Center for Health Services Research Brandenburg Medical School Theodor Fontane Rüdersdorf Germany; 6 Faculty of Health Sciences Brandenburg Brandenburg Medical School Theodor Fontane Potsdam Germany; 7 Verein zur Förderung der Rheumatologie e.V. Würselen Germany; 8 RheumaDatenRhePort (rhadar) Planegg Germany; 9 Medizinisches Versorgungszentrum Stolberg Stolberg Germany; 10 Klinik für Internistische Rheumatologie Rhein-Maas Klinikum Würselen Germany; 11 Rheumatologische Schwerpunktpraxis Drs. Kleinert, Rapp, Ronneberger, Schuch u. Wendler Erlangen Germany; 12 Department of Rheumatology University Hospital Düsseldorf Medical Faculty of Heinrich Heine University Düsseldorf Germany; 13 Institut Universitaire de France Paris France; 14 LabCom Telecom4Health Orange Labs & Université Grenoble Alpes CNRS, Inria, Grenoble INP-UGA Grenoble France; 15 MVZ für Rheumatologie Dr. Martin Welcker GmbH Planegg Germany; 16 Division of Rheumatology Klinikum Nürnberg Paracelsus Medical University Nürnberg Germany

**Keywords:** symptom checker, artificial intelligence, eHealth, diagnostic decision support system, rheumatology, decision support, decision, diagnostic, tool, rheumatologists, symptom assessment, resources, randomized controlled trial, diagnosis, decision support system, support system, support

## Abstract

**Background:**

The diagnosis of inflammatory rheumatic diseases (IRDs) is often delayed due to unspecific symptoms and a shortage of rheumatologists. Digital diagnostic decision support systems (DDSSs) have the potential to expedite diagnosis and help patients navigate the health care system more efficiently.

**Objective:**

The aim of this study was to assess the diagnostic accuracy of a mobile artificial intelligence (AI)–based symptom checker (Ada) and a web-based self-referral tool (Rheport) regarding IRDs.

**Methods:**

A prospective, multicenter, open-label, crossover randomized controlled trial was conducted with patients newly presenting to 3 rheumatology centers. Participants were randomly assigned to complete a symptom assessment using either Ada or Rheport. The primary outcome was the correct identification of IRDs by the DDSSs, defined as the presence of any IRD in the list of suggested diagnoses by Ada or achieving a prespecified threshold score with Rheport. The gold standard was the diagnosis made by rheumatologists.

**Results:**

A total of 600 patients were included, among whom 214 (35.7%) were diagnosed with an IRD. Most frequent IRD was rheumatoid arthritis with 69 (11.5%) patients. Rheport’s disease suggestion and Ada’s top 1 (D1) and top 5 (D5) disease suggestions demonstrated overall diagnostic accuracies of 52%, 63%, and 58%, respectively, for IRDs. Rheport showed a sensitivity of 62% and a specificity of 47% for IRDs. Ada’s D1 and D5 disease suggestions showed a sensitivity of 52% and 66%, respectively, and a specificity of 68% and 54%, respectively, concerning IRDs. Ada’s diagnostic accuracy regarding individual diagnoses was heterogenous, and Ada performed considerably better in identifying rheumatoid arthritis in comparison to other diagnoses (D1: 42%; D5: 64%). The Cohen κ statistic of Rheport for agreement on any rheumatic disease diagnosis with Ada D1 was 0.15 (95% CI 0.08-0.18) and with Ada D5 was 0.08 (95% CI 0.00-0.16), indicating poor agreement for the presence of any rheumatic disease between the 2 DDSSs.

**Conclusions:**

To our knowledge, this is the largest comparative DDSS trial with actual use of DDSSs by patients. The diagnostic accuracies of both DDSSs for IRDs were not promising in this high-prevalence patient population. DDSSs may lead to a misuse of scarce health care resources. Our results underscore the need for stringent regulation and drastic improvements to ensure the safety and efficacy of DDSSs.

**Trial Registration:**

German Register of Clinical Trials DRKS00017642; https://drks.de/search/en/trial/DRKS00017642

## Introduction

Symptoms caused by inflammatory rheumatic diseases (IRDs) are often unspecific and difficult to correctly interpret for patients [1] and even for experienced rheumatologists [2]. This diagnostic complexity frequently results in significant delay [3], which can diminish treatment efficacy and lead to progressive damage [4].

To address these challenges, a variety of freely available, patient-centered diagnostic decision support systems (DDSSs) have emerged and are increasingly being used by the general public [5] and patients with IRDs [6]. These DDSSs offer disease suggestions and advice for action within a few minutes and without any health care provider contact.

Rheport [7] is a web-based rheumatology referral system used in Germany to automatically triage appointments of new patients according to IRD probability [8,9]. To date, Rheport has been used to schedule more than 3000 appointments [10]. Ada [11], an artificial intelligence (AI)–based chatbot, is one of the most promising DDSS currently available. Multiple case-vignette studies showcased its high diagnostic accuracy [5,12], and more than 30 million symptom assessments have been completed in 130 countries [13].

Despite the expanding usage, little evidence is available regarding the accuracy of DDSSs in rheumatology [14-16]. To our knowledge, Rheport and Ada are among the most widely used DDSSs in rheumatology within Germany [10,13]. However, a direct comparative study between these 2 systems has yet to be conducted. Therefore, the aim of this analysis was to evaluate the diagnostic capability of Ada and Rheport in identifying IRDs.

## Methods

### Study Design and Participants

The study design for this pragmatic, prospective, multicenter, crossover randomized controlled trial (German Register of Clinical Trials DRKS00017642) has been described elsewhere in detail [9]. Results are presented according to the CONSORT-EHEALTH checklist (Multimedia Appendix 1) [17].

Adult patients with musculoskeletal symptoms who had been referred for the first time to 3 recruiting rheumatology outpatient clinics with a suspected diagnosis of an IRD were consecutively included in this study. Participants were instructed to enter the required data into both Ada and Rheport while waiting for their scheduled appointment with the rheumatologist. Assistance from support staff was available if needed. Patients were randomized 1:1 by computer-generated block randomization into group 1 (first Ada, then Rheport) or group 2 (first Rheport, then Ada), with each block comprising 100 patients. This crossover design was chosen to mitigate potential bias from a priming effect, where completing the first DDSS could influence responses to the second DDSS without the patient’s awareness. For instance, a priming effect was previously observed where participants who answered questions about their religiosity before reporting their alcohol consumption indicated fewer drinks on peak drinking occasions [18]. A designated project manager, uninvolved in the recruitment process, was responsible for assigning patients to the intervention arms. The statistician was kept blinded for group allocation. Assistance from the study personnel was available for DDSS completion when needed.

### Ethical Considerations

The study was approved by the ethics committee of the medical faculty of the University of Erlangen-Nürnberg, Germany (106_19 Bc), and was conducted in compliance with the Declaration of Helsinki. All patients provided written informed consent.

### The DDSSs

Rheport [7] comprises a static 23-item questionnaire designed to assess symptoms and generate an expert-derived weighted sum score [8,9]. Median completion time was 8 minutes [19]. A higher sum score correlates with an increased probability of an IRD. Rheumatologists utilizing this system can allocate slots for automatic patient scheduling. Based on the calculated IRD probability, patients are offered available appointments categorized into 4 urgency levels. Patients with scores below 1 are considered unlikely to have an IRD and do not receive an appointment. Those with a minimum score of 1 are considered likely to have an IRD and are enabled to book an appointment. The urgency levels are categorized as follows: patients with scores between 1 and 2.4 are considered intermediate, patients with scores between 2.4 and 4 are considered urgent, and patients with scores exceeding 4 are considered very urgent. Patients in the very urgent category should ideally receive appointments within 1 week [19]. Upon the acceptance of a proposed appointment by a patient, the rheumatologist is notified and provided with a structured summary report of the questionnaire. There were no changes to the Rheport algorithm during the study period.

Ada [11] is a native app and certified medical product, designed to cover a broad spectrum of symptoms and diseases. Programmed as a chatbot, Ada mimics traditional history taking by initially requesting basic health information, such as sex and age, followed by current symptoms. Based on these responses, the app generates individualized follow-up questions. Ada’s diagnostic suggestions are driven by a Bayesian network that is continuously updated [20]. Upon the completion of symptom querying (median time: 7 minutes [19]), a summary report is generated, including (1) a summary of present, absent, and uncertain symptoms; (2) up to 5 disease suggestions with the corresponding probabilities, triage recommendations (eg, call an ambulance), and symptom relevance; and (3) access to basic information about the suggested diseases. Ada was regularly updated along the course of the study to ensure functionality.

### Outcome

The primary end point of the study was concordant detection of any IRD diagnosis (including, eg, rheumatoid arthritis or systemic lupus erythematosus) by the DDSSs and the gold standard, that is, the rheumatologist’s final diagnosis, reported on the discharge summary report and adjudicated by the attending head physician of the local rheumatology department. For Rheport, the detection of an IRD by the DDSS was defined as a sum score of 1 or higher. Regarding Ada, we analyzed whether there was an IRD diagnosis and whether the correct diagnosis was listed as the top diagnosis (Ada top 1 [D1]) or was listed at all among all suggested diagnoses (Ada top 5 [D5]).

### Statistical Analysis

Descriptive characteristics are presented as median and IQR for interval data and as absolute (n) and relative frequency (percentage) for nominal data. The minimum necessary sample size for this study was 122 in order to detect a specificity and sensitivity of at least 70% for Ada or Rheport, with a type I error of 4.4% and type II error of 19% using a 1-sample test [21] against a benchmark accuracy of 50% based on a previous evaluation of DDSSs [14]. Operating characteristics of Ada and Rheport for a diagnosis of rheumatic disease was evaluated using sensitivity, specificity, negative predictive value, positive predictive value, and overall accuracy with respective 95% CIs. This evaluation was done both separately and for the combined use of the DDSSs. The agreement between the DDSSs was evaluated using the Cohen κ statistic, with values ≤0 indicating no agreement, 0.01-0.20 indicating none to slight agreement, 0.21-0.40 indicating fair agreement, 0.41-0.60 indicating moderate agreement, 0.61-0.80 indicating substantial agreement, and 0.81-1.00 indicating almost perfect agreement [22]. We evaluated the cumulative proportion of correct diagnoses using Ada with exact CIs. We used a binomial regression with a log-link function to calculate the risk ratio for correct identification of any IRD by Rheport in comparison to Ada when the respective DDSS was used first and when it was used after the crossover. We preferred this method over logistic regression since the interpretation of risk ratios is more intuitive than that for odds ratios with high-prevalence binary outcomes. All analyses were conducted using the open-source R software (version 4.1.0; R Foundation for Statistical Computing) running under RStudio (version 1.4.1103; RStudio).

## Results

### Participants

**Figure 1:**
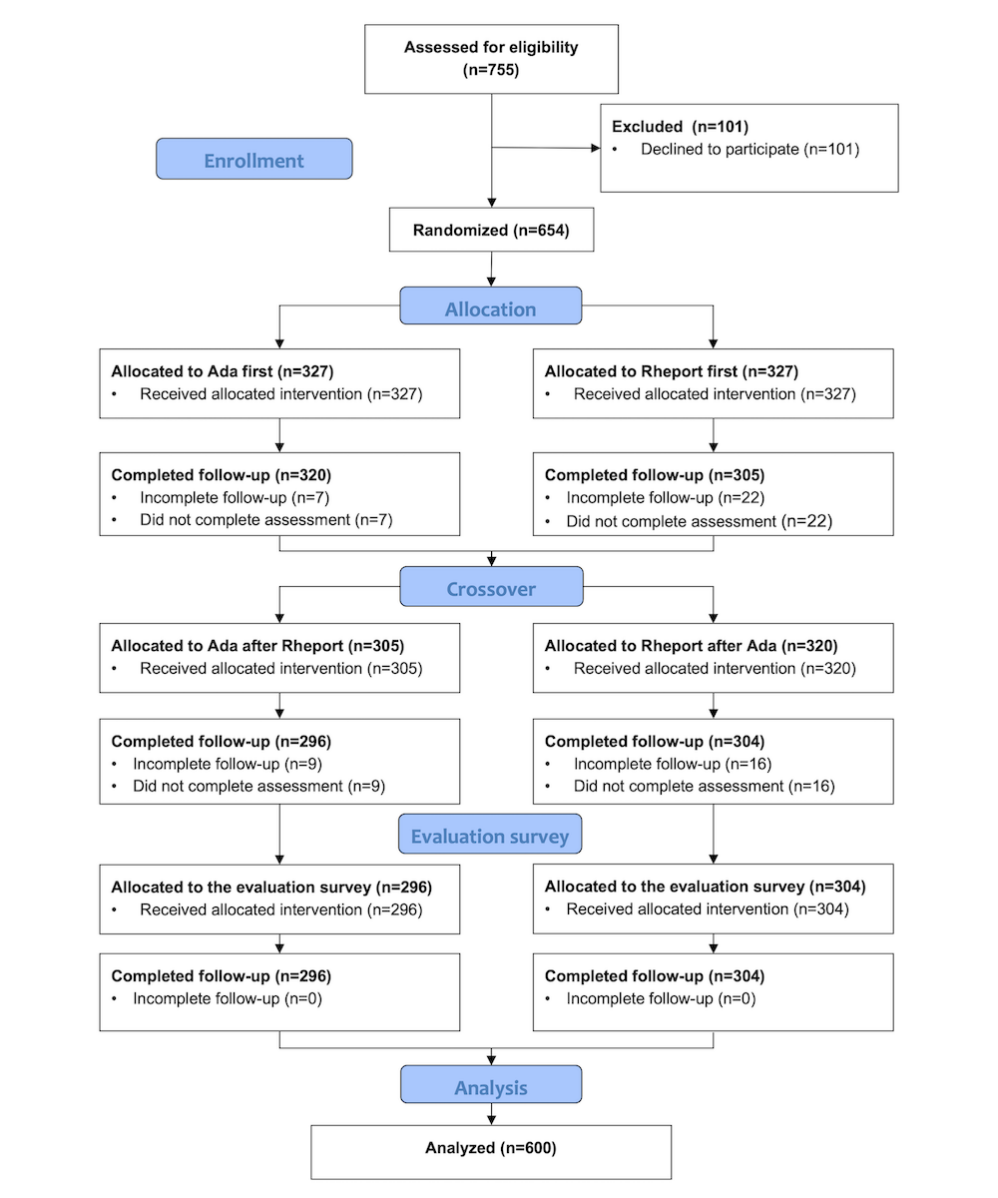
Patient flow diagram.

A total of 755 consecutive patients were approached between September 2019 and April 2021, of whom 654 (87%) agreed to participate and 600 (79.4%) were included in the analysis presented (Figure 1). The participation exceeded the minimal sample size calculation, since patients were very eager to participate and considered the study a welcome distraction during the waiting time for their appointment. Overall, 35.7% (214/600) of the patients were diagnosed with an IRD based on physicians’ judgment.

The demographic characteristics of the patients are displayed in Table 1, and the physicians’ final diagnoses are presented in Table 2.

**Table 1:** Patient demographic characteristics according to the physicians’ final diagnosis, reported on the discharge summary report.

Demographics	Final diagnosis		Total sample (n=600)
	IRD^a^ (n=214)	Non-IRD (n=386)	
Age (years), median (IQR)	53.0 (38.0-64.0)	51.0 (37.0-59.0)	52.0 (37.0-61.0)
Sex (female), n (%)	131 (61.2)	287 (74.4)	418 (69.7)
Tender joint count 28, median (IQR)	1.0 (0.0-3.0)	0.0 (0.0-1.0)	0.0 (1.0-2.0)
Swollen joint count 28, median (IQR)	0.0 (0.0-2.0)	0.0 (0.0-0.0)	0.0 (0.0-0.0)
VAS^b^ patient global (mm), median (IQR)	35.0 (9.5-60.0)	40.0 (10.0-60.0)	40.0 (10.0-60.0)
ESR^c^ (mm/h), median (IQR)	13.0 (6.0-25.0)	8.0 (5.0-13.0)	9.0 (5.0-17.0)
CRP^d^ (mg/L), median (IQR)	5.0 (5.0-8.8)	5.0 (3.1-5.0)	5.0 (3.2-5.0)
DAS28^e^ (CRP), median (IQR)	3.0 (2.2-3.9)	2.2 (1.7-2.9)	2.5 (1.8-3.2)
RF^f^ (positive), n/N (%)	36/196 (18.4)	34/350 (9.7)	70/546 (11.7)
ACPA^g^ (positive), n/N (%)	20/192 (10.4)	5/336 (1.5)	25/528 (4.2)

^a^IRD: inflammatory rheumatic disease.

^b^VAS: visual analogue scale.

^c^ESR: erythrocyte sedimentation rate.

^d^CRP: C-reactive protein.

^e^DAS28: disease activity score 28.

^f^RF: rheumatoid factor.

^g^ACPA: anticitrullinated protein antibodies.

**Table 2:** Patients according to diagnostic categories.

Diagnostic categories		Patients (n=600), n (%)
**IRD^a^**		214 (35.7)
	Axial spondyloarthritis	31 (5.2)
	Connective tissue disease	22 (3.7)
	Crystal arthropathies	8 (1.3)
	Peripheral spondyloarthritis	3 (0.5)
	Polymyalgia rheumatica	16 (2.7)
	Psoriatic arthritis	31 (5.2)
	Rheumatoid arthritis	69 (11.5)
	Undifferentiated arthritis	19 (3.2)
	Vasculitis	8 (1.3)
	Other IRD	7 (1.2)
**Non-IRD**		386 (64.3)
	Osteoarthritis	71 (11.8)
	Fibromyalgia	37 (6.2)
	Other noninflammatory, unclear diagnosis	278 (46.3)

^a^IRD: inflammatory rheumatic disease.

### Diagnostic Accuracy of Ada and Rheport

**Figure 2:**
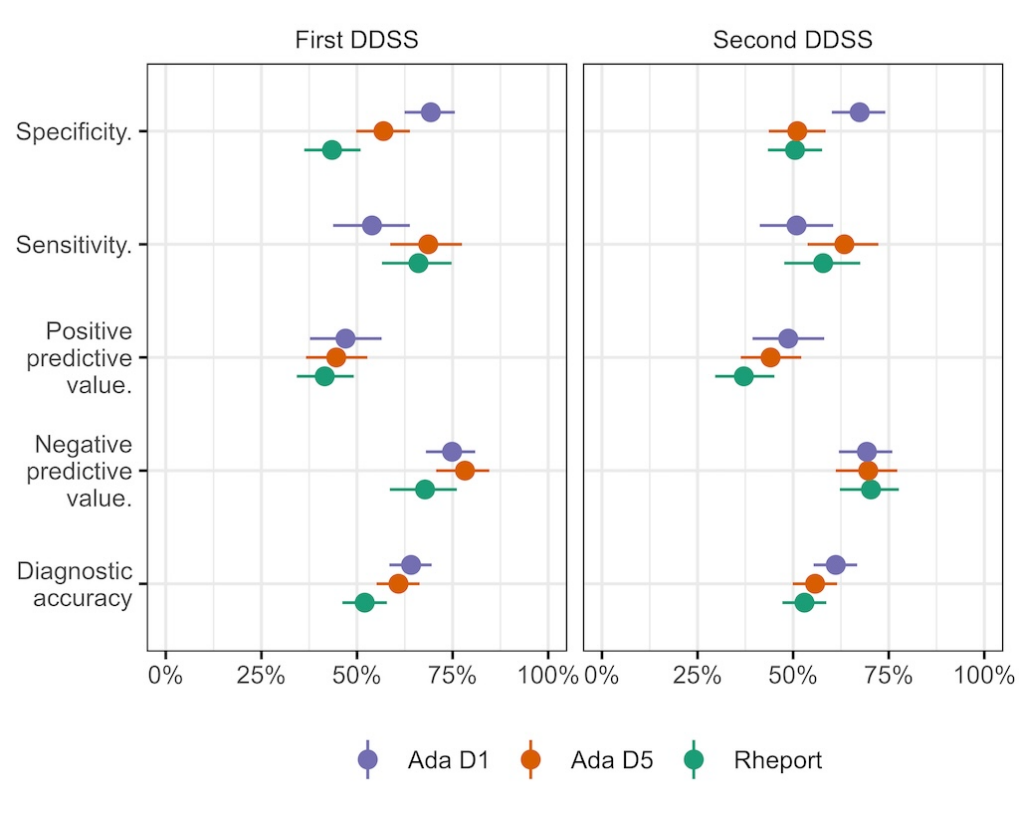
Diagnostic properties of Ada and Rheport regarding IRDs according to the order of usage. Ada D1: Ada's top diagnosis; Ada D5: Ada's top 5 suggestions; IRD: inflammatory rheumatic disease; DDSS: diagnostic decision support system.

Rheport showed an overall sensitivity of 62% and a specificity of 47% for IRDs (Figure 2). Ada’s D1 and D5 disease suggestions showed a sensitivity of 52% and 66%, respectively, and a specificity of 68% and 54%, respectively, concerning IRDs (Table 3). The odds ratio for Rheport correctly suggesting a rheumatic disease diagnosis in comparison to Ada D5 as the first used DDSS was 0.89 (95% CI 0.83-0.97). When the initial DDSS was Ada, the accuracy of Ada D5 was 61% (95% CI 55%-66%) and the accuracy of Rheport was 53% (95% CI 47%-59%), whereas after the crossover, this odds ratio was 0.98 (95% CI 0.91-1.06) with corresponding accuracies of Ada D5 at 56% (95% CI 50%-61%) and of Rheport at 52% (95% CI 46%-58%).

**Figure 3:**
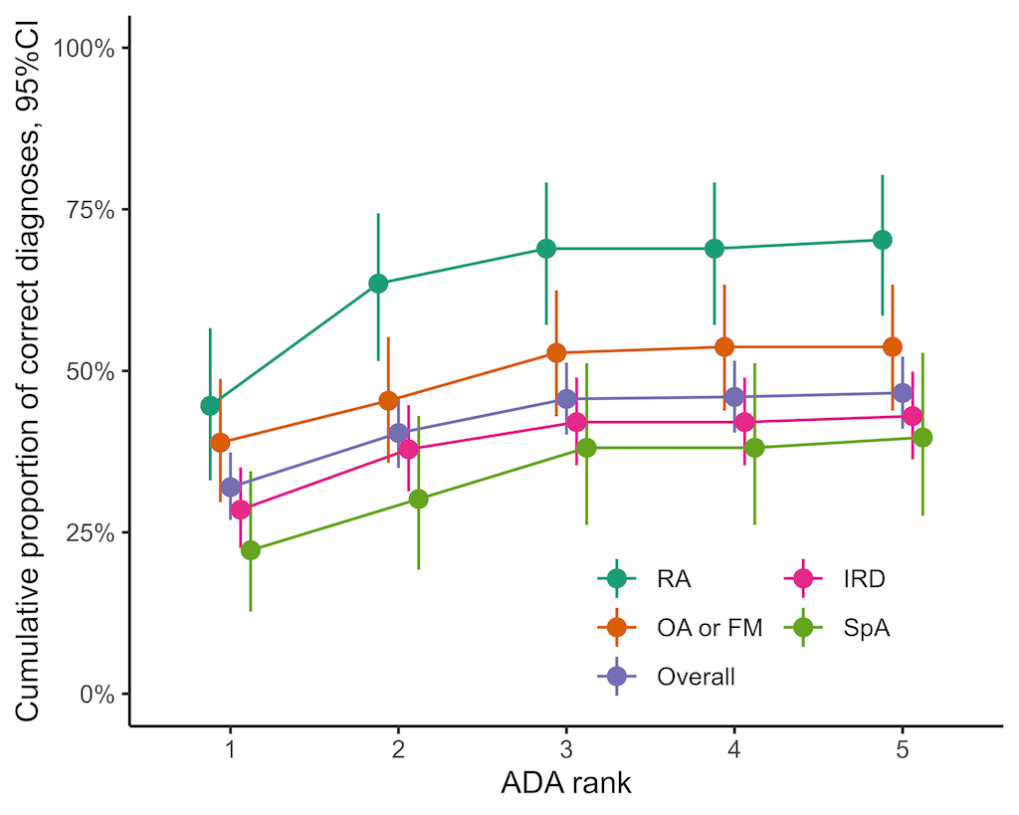
Cumulative overall diagnostic accuracy of Ada for selected diseases according to diagnostic rank. FM: fibromyalgia; IRD: inflammatory rheumatic diseases; OA: osteoarthritis; overall: all patients with a final medical diagnosis; RA: rheumatoid arthritis; SpA: spondyloarthritis.

Ada’s diagnostic accuracy regarding individual diagnoses was heterogenous. Ada suggested the correct diagnosis of as top suggestion (Ada D1) in 42% (29/69) of patients with rheumatoid arthritis, and the correct diagnosis was suggested overall (Ada D5) in 64% (44/69); moreover, the first suggestion of Ada (Ada D1) was correct in 22% (14/65) of patients with spondyloarthritis (including axial spondyloarthritis, peripheral spondyloarthritis, and psoriatic arthritis), and the correct diagnosis was suggested overall (Ada D5) in 38% (25/65). The findings suggest that Ada performed considerably better in identifying rheumatoid arthritis in comparison to other diagnoses (see Figure 3).

**Table 3:** Sensitivity, specificity, PPV^a^, NPV^b^, and overall accuracy of Ada and Rheport for the diagnosis of inflammatory rheumatic diseases including 95% CIs.

Characteristic		Diagnostic decision support system		
		Rheport	Ada D1^c^	Ada D5^d^
**Total** **sample**				
	Sensitivity (%; 95% CI)	62 (55-69)	52 (45-59)	66 (59-72)
	Specificity (%; 95% CI)	47 (42-52)	68 (63-73)	54 (49-59)
	PPV (%; 95% CI)	39 (34-45)	48 (41-54)	44 (39-50)
	NPV (%; 95% CI)	69 (63-75)	72 (67-77)	74 (69-79)
	Accuracy (%; 95% CI)	52 (48-57)	63 (59-67)	58 (54-62)
**Ada first**				
	Sensitivity (%; 95% CI)	58 (48-68)	54 (44-64)	69 (59-77)
	Specificity (%; 95% CI)	50 (43-58)	69 (62-76)	57 (50-64)
	PPV (%; 95% CI)	37 (30-45)	47 (38-56)	45 (37-53)
	NPV (%; 95% CI)	70 (62-78)	75 (68-81)	78 (71-85)
	Accuracy (%; 95% CI)	53 (47-59)	64 (58-70)	61 (55-66)
**Rheport first**				
	Sensitivity (%; 95% CI)	66 (57-75)	51 (41-60)	63 (54-72)
	Specificity (%; 95% CI)	43 (36-51)	67 (60-74)	51 (44-59)
	PPV (%; 95% CI)	42 (34-49)	49 (39-58)	44 (36-52)
	NPV (%; 95% CI)	68 (59-76)	69 (62-76)	70 (61-77)
	Accuracy (%; 95% CI)	52 (46-58)	61 (55-67)	56 (50-61)

^a^PPV: positive predictive value.

^b^NPV: negative predictive value.

^c^Ada D1: using Ada’s top suggestion only.

^d^Ada D5: using all suggestions provided by Ada.

### Agreement Between Rheport and Ada

The Cohen κ statistic of Rheport for agreement on any rheumatic disease diagnosis with Ada D1 was 0.15 (95% CI 0.08-0.18) and with Ada D5 was 0.08 (95% CI 0.00-0.16), indicating poor or nonexistent agreement for the presence of any rheumatic disease between the 2 DDSSs.

## Discussion

### Principal Findings

This prospective, multicenter randomized controlled trial investigated the diagnostic accuracy of 2 DDSSs regarding IRDs. Overall, the diagnostic accuracy of both DDSSs was limited. Rheport was less likely to correctly identify any IRD when used as the first DDSS; this could not be reproduced when it was used as the second DDSS. The diagnostic accuracy was comparably low with both tools, although Ada is an AI-based chatbot whereas Rheport is built on a simple weighted-sum-score questionnaire and expert opinions. The low negative predictive values of both Ada and Rheport suggest frequent errors when used as rheumatic screening tools. The low diagnostic accuracy is all the more alarming, as this study population already had a higher pretest probability. Patients were highly preselected with physicians explicitly only referring patients with a suspected IRD. Overall, these final results confirm the low diagnostic accuracy of both DDSSs observed in the interim analysis, which included 164 patients from a single participating center [9]. This study also confirms the case-dependent variations in the diagnostic accuracy of Ada, which was the highest for rheumatoid arthritis, in line with the results of a previous vignette-based study [23,24].

Two previous studies have demonstrated the strong user dependence of Ada’s diagnostic accuracy [23,25]. The low DDSS accuracy for IRDs is in line with previous results from smaller studies. In a pilot study, Powley et al [15] showed that only 19% of patients with IRDs were correctly identified. Similarly, Proft et al [26] recently showed that only 19% of patients using an axial spondyloarthritis self-referral tool were actually correctly diagnosed. A general reason for the low diagnostic accuracy of symptom-based DDSSs in rheumatology could be the lack of available information compared to the physician. Ehrenstein et al [2] previously demonstrated that the diagnostic accuracy of experienced rheumatologists regarding correct identification of IRDs solely based on medical history was only 14%. The low accuracy of DDSSs poses substantial challenges, as inaccurate diagnoses can cause misutilization of scarce health care resources, anxiety among patients, and frustration among health care professionals. Complementing subjective symptom descriptions by adding objective laboratory values obtained via self-sampling [27,28] could improve the accuracy of DDSS suggestions while preserving remote care advantages. Furthermore, the application of machine learning has proven effective in improving the diagnostic accuracy of the current Rheport algorithm, highlighted by an increase in the area under the receiver operating characteristic curve from 0.534 to 0.737 [29]. The top 5 most significant features identified by the best-performing logistic regression model for IRD classification included finger joint pain, elevated inflammatory marker levels, the presence of psoriasis, symptom duration, and female sex. Additionally, the integration of large language models such as ChatGPT could significantly improve the DDSS performance [30]. In a recent study, we demonstrated that ChatGPT achieved diagnostic accuracy for rheumatic diseases comparable to that of experienced rheumatologists when both were provided with identical summary reports generated by patients using Ada. Additionally, we believe that usability may be improved by incorporating the free-text input and voice-enabled features of ChatGPT. A scoping review has highlighted the poor diagnostic accuracy, lack of evidence, and absence of regulation for patient-facing DDSSs [31]. To address these issues, we echo the calls for the implementation of stricter regulatory frameworks, certification procedures, and ongoing monitoring to close these regulatory gaps [31].

### Limitations

A main limitation of the study is the fact that patients were already screened by referring physicians, causing a much higher a priori chance of having an IRD. Furthermore, help from assisting personnel was available if needed for DDSS completion. To our knowledge, this is the largest comparative DDSS trial with real patients; however, the results of this study are not automatically transferable to other disciplines, languages, patient groups, and DDSSs. We therefore call for future studies involving real patients to build more solid evidence.

### Conclusions

Overall, the diagnostic accuracy and agreement of both DDSSs regarding IRDs were limited. Improvements are needed to ensure DDSS safety and efficacy. The results suggest that physicians and the complex process of establishing a medical diagnosis cannot be replaced by an algorithm-based or AI-based DDSS. Future studies are needed to evaluate the generalizability of our findings.

## Appendix

Multimedia Appendix 1 CONSORT eHEALTH checklist (V 1.6.1).

## Abbreviations

AI: artificial intelligence
D1: top 1
D5: top 5
DDSS: diagnostic decision support system
IRD: inflammatory rheumatic disease
